# Salivary Brain-Derived Neurotrophic Factor and Cortisol Associated with Psychological Alterations in University Students

**DOI:** 10.3390/diagnostics14040447

**Published:** 2024-02-18

**Authors:** María Luisa Ballestar-Tarín, Vanessa Ibáñez-del Valle, Mayra Alejandra Mafla-España, Rut Navarro-Martínez, Omar Cauli

**Affiliations:** 1 Department of Nursing, University of Valencia, 46010 Valencia, Spain; m.luisa.ballestar@uv.es (M.L.B.-T.); maymaes@alumni.uv.es (M.A.M.-E.); rut.navarro@uv.es (R.N.-M.); 2 Nursing Care and Education Research Group in (GRIECE) GIUV 2019-456, Department of Nursing, University of Valencia, 46010 Valencia, Spain; 3 Frailty Research Organized Group (FROG), University of Valencia, 46010 Valencia, Spain; 4 Department of Hematology, University General Hospital, 46014 Valencia, Spain

**Keywords:** students, depressive symptoms, brain-derived neurotrophic factor, cortisol, stress, sleep, salivary biomarkers

## Abstract

Introduction: Recent evidence reported mental health issues in university students such as anxiety and depressive symptoms and poor sleep quality. Decreased plasma brain-derived neurotrophic factor (BDNF) levels have been proposed as a biomarker of depressive symptoms, whereas cortisol levels are an index of energy mobilization and stress and have been linked to sleep quality. Given that salivary biomarkers represent an interesting new field of research, the aim of this cross-sectional study was to evaluate salivary BDNF and cortisol levels in university students to assess whether they have associations with psychological disturbances such as anxiety and depressive symptoms, sleep quality, and stress level. Methods: Salivary BDNF and cortisol levels were measured by specific immunoassays in 70 students whose mental health was also evaluated on the same day through the evaluation of anxiety and depression symptoms (Goldberg scale), sleep quality (Pittsburg Sleep Quality Index and Athens Insomnia Scale), and stress (self-perceived stress scale) and healthy lifestyle habits (alcohol consumption, smoking, regular exercise, and body mass index) were also measured. Multivariate regression analyses were performed in order to identify the strengths of associations between psychological alterations and the concentrations of BDNF, cortisol, and other variables. Results: Salivary BDNF levels were significantly higher in students with more depressive symptoms, whereas no significant differences were found for cortisol levels. When performing the binary logistic regression model, BDNF levels are included as a predictor variable for a high-depressive-symptoms burden (*p* < 0.05). Students with worse sleep quality on the Pittsburg Scale had higher cortisol levels (*p* < 0.05). The subdomains of sleep latency and sleep medication were those significantly associated with salivary cortisol levels in logistic regression analyses (OR = 15.150, *p* = 0.028). Sleep medication only appeared to be related to cortisol levels (OR = 185.142, *p* = 0.019). Perceived stress levels and anxiety symptoms were not associated with BDNF or cortisol levels. Conclusions: BDNF could play a key role in the pathophysiology of mood-related disorders, and elevation of its peripheral levels could contribute to protecting neurons from the development of mental illness. Higher salivary cortisol levels measured in the morning are accompanied by poorer sleep quality. More research is needed, focusing on salivary biomarkers of disorders related to depressive symptoms and poor sleep quality as a potential tool for the diagnosis and prevention of mental illness.

## 1. Introduction

The mental health and well-being of university students is currently considered a growing public health problem [1,2,3,4,5]. Globally, studies in different samples of university students have identified a moderate to high prevalence of depression and anxiety in this population. In the study by Shamsuddin et al. [6], conducted with Malaysian university students, the results showed that 27.5% of the students had moderate depression and 9.7% had severe or extremely severe depression. Regarding anxiety, 34% had moderate anxiety and 29% had severe or extremely severe anxiety. The study by Wong et al. [7] found that 21% and 41% of Hong Kong university students had moderate or higher levels of depression and anxiety, respectively. Rates of anxiety and depressive symptoms were also high in the study by Bahhawi et al. [8], which found a prevalence of moderate depression of 53.6% in the sample studied (students in Saudi Arabia) and a prevalence of anxiety of 65.7%. In other studies, such as the study conducted by Sandal et al. [9] with students from Punjab University, the overall prevalences of depression and anxiety were also high, at 59.2% and 86.5%, respectively.

Although mental health problems are highly prevalent in the general population in Spain [10], there are few studies on the mental health of Spanish university students [11]. In the study by Fernández-Rodríguez et al. [12], the results showed a high prevalence of anxiety and depression in students in all branches of knowledge at the University of Oviedo (Oviedo, Spain). Among the participants, 44.7% presented levels of emotional distress indicative of anxiety and 13.5% of depression. Ramón-Arbués et al. [11] conducted a study on the prevalence of anxiety, depression, and stress symptoms and their associated factors in a sample of Spanish university students from various bachelor’s degree programs. Their results showed a moderate prevalence of symptoms of depression (18.4%), anxiety (23.6%), and stress (34.5%) in the population studied. Different studies claim that the COVID-19 pandemic has affected the mental health of young people, worsening their emotional well-being [12]. Young adults and adolescents have been particularly vulnerable to the mental health consequences of the COVID-19 pandemic and its lockdown [13,14]. A meta-analysis [15], including 13,247 nursing students, identified a prevalence of depression of 52% and anxiety of 32%, in addition to other health problems. In Spain, the study conducted by Ibáñez et al. [16] also found high percentages of students with anxiety and depression symptoms among nursing students, i.e., 45.3% and 46.4%, respectively.

According to the neurotrophic hypothesis, it is likely that stress and depression are associated with a deficit of neurotrophins, leading to neuronal atrophy and cell loss in key limbic areas and the prefrontal cortex. Antidepressant treatments can block or reverse these effects [17,18,19]. Special attention has been paid to brain-derived neurotrophic factor (BDNF), a neurotrophin that plays a key role as an allostatic mediator that supports functions such as memory and learning, as well as continuous adaptations to environmental perturbations [20]. Several recent studies have shown that peripheral (serum and/or plasma) levels of BDNF are lower in patients suffering from mood disorders during manic/mixed and depressive episodes than in matched healthy controls and that effective treatments are capable of normalizing them [21,22,23,24,25,26,27,28]. These data suggest that peripheral BDNF levels are potential biomarkers of mood episodes (depressive and manic/mixed), as well as predictors of treatment efficacy. However, BDNF levels in saliva [29] have not been studied, and their relationships with depressive symptoms even less so.

Meanwhile, blood cortisol levels are a widely accepted index of energy mobilization and stress levels [30]. A meta-analysis of cortisol awakening responses and psychosocial factors revealed that levels of chronic psychological stress, such as work stress and general life stress, were associated with increased cortisol awakening responses [31]. However, studies associating job stress with cortisol levels have yielded inconsistent results, with some studies reporting no association [32], whereas others describe a link between low levels of cortisol and high work stress [33]. In healthy humans, Begliuomini et al. [34] found a positive correlation between blood BDNF and cortisol levels. Both BDNF and cortisol were significantly higher in the morning than in the evening, showing a parallel diurnal rhythm.

The current literature lacks data for BDNF levels in students and mental health, such as symptoms of anxiety and depression, insomnia, and stress, and it seems crucial to investigate markers of neuroplasticity, such as peripheral BDNF levels and cortisol that can be detected non-invasively in saliva samples. Since different studies have linked peripheral BDNF concentration in blood with depressive symptoms [27,35,36,37], we hypothesized that salivary BDNF levels could be associated with depressive symptoms and other psychological variables related to depressive symptoms such as stress, anxiety, and sleep quality in university students. The objective of this study was, therefore, to measure the levels of salivary BDNF and cortisol in university students and to investigate the possible correlations with healthy-lifestyle variables and symptoms of anxiety, depression, stress, and insomnia that have been shown to be altered in a large proportion of this population.

## 2. Material and Methods

### 2.1. Study Design and Population

A cross-sectional observational study was conducted between November 2020 and February 2021. The sample consisted of university students on the nursing degree course at the University of Valencia, Valencia (Spain). The students were asked to self-complete a questionnaire that included sociodemographic variables (age, sex, marital status, cohabitation, and employment status) as well as lifestyle (physical activity, smoking, and alcohol) and health data (chronic diseases and perceived health status), the Athens Insomnia Scale, the Pittsburg Sleep Quality Index, the Cohen Perceived Stress Questionnaire, and the Goldberg Anxiety and Depression Scale. Alcohol consumption was measured using AUDIT-C3. A saliva sample was also collected to measure salivary cortisol and BDNF.

### 2.2. Alcohol Consumption

The AUDIT was developed by the World Health Organization (WHO) as a simple screening method for excessive alcohol consumption. The AUDIT-C3 is a rapid screening test for heavy alcohol consumption. It consists of 3 questions that score from 0 to 4. The cut-off point for males is 6 points, and for females it is 4 points [38].

### 2.3. Self-Perceived Stress

Perceived stress was assessed using Cohen’s Perceived Stress Scale (PSS), which has been validated in Spanish. The PSS is the best-known instrument for measuring perceived psychological stress, i.e., the degree to which everyday life situations are valued as stressful. This scale is composed of ten items that are used to assess how often the respondent has felt a certain way during the last month. The responses are scored 0 (never), 1 (almost never), 2 (sometimes), 3 (quite a lot), or 4 (very often). However, items 4, 5, 7, and 8 are scored in reverse or inverted form. The total score ranges from 0 to 40 points, where higher scores indicate a higher degree of perceived stress. Without having established a cut-off point, the third quartile has been used to dichotomize the scale, thus establishing two categories: 0 (without stress symptoms) and 1 (with stress symptoms).

### 2.4. Quality of Sleep

#### 2.4.1. Athens Insomnia Scale

This scale was developed by Soldados et al. [39] to evaluate insomnia according to ICD-10 criteria. It consists of 10 items, with Likert-type responses ranging from 0 (no problem) to 3 (serious problem), and the overall score ranges from 0 to 30 points. Participants respond according to whether they have experienced those difficulties at least three times a week during the last month. The cut-off point is set at 6 points, so a score of 6 points or more on the Athens Insomnia questionnaire is considered poor sleep quality, and a score lower than 6 indicates normal sleep. Two subdimensions can be identified on the Athens scale. The first refers to difficulty sleeping, and the second to daytime sleepiness. The Athens scale has been translated and validated into the Spanish language in two studies [40,41] performed on young adults of similar ages, i.e., 20–30 years old. The cut-off used was the same as used in the Greek population [39]. Many other studies have used the same cut-off in Spanish university students [42,43,44]. The cut-off of the Athens scale is based on the criteria for frequency and duration of sleep difficulties corresponding to ICD-10 [45] criterion B for the diagnosis of non-organic insomnia.

#### 2.4.2. Pittsburgh Scale

The Pittsburgh Sleep Quality Index (PSQI) is a self-administered questionnaire that provides an overall rating of sleep quality. The PSQI contains a total of 19 items. These 19 items combine to form seven subdomains with corresponding scores (subjective sleep quality, sleep latency, sleep duration, sleep efficiency, sleep disturbances, sleep medication use, and daytime dysfunction), each of which shows a range between 0 and 3 points. A score of “0” indicates no problem, while a score of “3” indicates a major problem within the area concerned. The scores for the seven areas are finally added together to give an overall score, which ranges from 0 to 21 points. “0” indicates ease of sleeping, and “21” indicates severe difficulty in all areas. A score of 5 is used to categorize between good (≤5) and poor sleepers (>5). To facilitate the interpretation of the analyses, each dimension has been dichotomized, and as such, no problem (0) includes categories 0 and 1 (no problem or a slight problem), and 1 includes categories 2 and 3 (a problem or a severe problem). The Pittsburgh Sleep Quality Index (PSQI) was validated with a Spanish population by Royuela & Fernánez [46]. These authors conducted a study in which they interviewed two different populations. As in the original version, a total score above 5 was taken as the cut-off point. Thus, those with scores of 5 were considered good sleepers and those with above 5 bad sleepers. A recent study by Carrión-Pantoja et al. [47] also used the PSQI to measure the prevalence of insomnia symptoms in a population of Spanish university students and considered a PSQI global score > 5 as the cut-off point for clinically problematic sleep.

### 2.5. Depressive and Anxiety Symptoms

The Goldberg Anxiety and Depression Scale, which has been validated in Spanish, was used to evaluate the level of anxiety or depression. This questionnaire not only orients the diagnosis towards anxiety or depression (or both in mixed cases) but also discriminates between them and quantifies their respective intensities. This scale consists of two subscales: one for anxiety and one for depression. Each of the subscales is composed of nine dichotomous response items (YES or NO) to determine whether the respondent has had any of the symptoms in the last two weeks. The cut-off points are four or more items or affirmative responses for the anxiety scale and two or more for the depression scale. The higher the score, the more severe the problem (the maximum possible score is 9 points for each of the subscales). Cut-off points have been proposed (≥4 for anxiety and ≥2 for depression) to consider “probable cases” [48,49]. Montón et al. [50] validated the Goldberg scale in the Spanish population in a sample of 444 patients aged 19 and over. The Goldberg scale has previously been used to assess anxiety and depression symptoms among college students [51,52], healthcare professionals [53], and the Spanish general population [54].

### 2.6. Measurement of BDNF and Cortisol

The participants were asked to refrain from smoking, eating, drinking, or undergoing oral hygiene procedures for at least 1 h before the sample collection. The saliva samples were always collected in the morning (between 10 and 11 a.m.) in order to minimize the effect of circadian rhythm production. The saliva samples were collected using the Salivette^®^ system (Sarstedt, Germany) for the assessment of salivary markers (BDNF and cortisol). The samples were collected in the classroom where the students volunteered. Each sample was centrifuged to remove mucins, insoluble material, and cellular debris, and the supernatant was aliquoted into Eppendorf tubes and frozen (−80 °C) until further analysis. The samples (100 μL) were brought to room temperature, and ELISA assays were performed in duplicate using the commercial Abcam High Sensitivity Human Elisa Kit for BDNF (ab212166) and for cortisol (ab154996) according to the manufacturer’s instructions. Changes in color intensity and absorbance at 450 nm and 490 nm were read using an ELISA microplate reader, and a standard curve was prepared by plotting absorbance readings of standards against their concentrations.

### 2.7. Statistical Analysis

A descriptive analysis of the sociodemographic variables was performed, showing the distribution of percentages for qualitative variables, and the mean and standard deviation for quantitative variables. Non-compliance with normality was tested using the Kolmogorov–Smirnov test. The Student’s *t*-test or the Mann–Whitney test was used for the comparison of means between quantitative variables, and the Chi-squared test was used for the comparison of proportions. Logistic regressions were used to produce a predictive model of emotional well-being (depression and insomnia) and BDNF and cortisol levels. Logistic regression can be used to simultaneously assess several factors presumably related in some way to the dependent variable. Logistic regression analysis allows us to obtain measures of association (odds ratios) for each variable adjusted for the others, and to detect possible interactions between them and the effect studied. First, logistic regression was performed to determine which among sociodemographic variables, lifestyle variables, and BDNF levels are related to depression. Another logistic regression analysis was performed to analyze the sociodemographic and lifestyle variables and cortisol levels related to insomnia. Statistical significance was established at *p* < 0.05 in all cases. Statistical analysis was performed using SPSS software (version 28.0, IBM Corp., Armonk, NY, USA).

## 3. Results

### 3.1. Sociodemographic Characteristics of the Study Sample

The sample consisted of 72 students from the Faculty of Nursing and Podiatry of the University of Valencia (79.2% women and 20.8% men). The mean age of the participants was 20 years (SD = 5.569). Seventy-five percent of the students were single, living mainly with their family (70.8%). 86.3% were full-time students and 13.9% work part-time or full-time. A total of 15.9% of the participants reported suffering from a chronic illness, and 59.4% stated that they regularly practice sports. The sample considers their health status to be 7.88 (SD = 1.22) with no differences between men and women (*p* > 0.05).

### 3.2. Alcohol and Tobacco Use

A total of 84.7% of the participants do not smoke, 4.2% smoke occasionally, and 11.1% smoke regularly. As regards alcohol consumption, only 12.5% said that they do not consume alcohol. On the other hand, 31.9% of the participants said that they had never consumed six or more drinks on one occasion, but 38.9% said that they did so every time they drank. According to the AUDIT-C questionnaire, 56.9% of the participants had a risky drinking pattern. Higher levels of consumption were recorded in women than in men (χ^2^ = 19.535, *p* < 0.05). The pattern of alcohol consumption is not related to playing sports (χ^2^ = 3.129, *p* = 0.120) or having a chronic disease (χ^2^ = 1.170, *p* = 0.336).

### 3.3. Sleep Quality

Sleep quality was assessed with two questionnaires: the Athens Insomnia Scale and the Pittsburgh Sleep Quality Index. The items in the Athens questionnaire assess different aspects of sleep. Sleep induction, sleep duration, daytime sleepiness, and sleep duration are the aspects with the worst scores. A total of 22.2% of the participants had a moderate problem with sleep induction, while 20.8% of the students indicated that their total sleep duration was markedly insufficient, and 16.7% described their daytime sleepiness as considerable. Based on the dichotomization of the Athens Insomnia Scale, 44.4% of the participants stated that they had poor sleep quality, while 55.6% said they had good sleep quality. No significant differences between men and women were observed (χ^2^ = 0.152, *p* = 0.697). The mean score was 5.42 (SD = 3.29). The prevalence of poor sleep quality with the Pittsburg quality index data was similar to that obtained with the Athens Insomnia Scale. If we consider a cut-off point of 5 as distinguishing between good and poor sleep quality according to PSQI analysis instructions, 59.7% of the participants had poor sleep quality compared to 40.3% who slept well. There were no significant differences between men and women (χ^2^ = 1.343, *p* = 0.247). The mean score on the sleep quality index was 6.75 (SD = 3.236). The components of the PSQI with the worst scores were daytime dysfunction, sleep latency, and sleep duration, which 36.1–41.7% of students considered to be fairly bad or very bad (Table 1).

### 3.4. Self-Perceived Stress

The mean score obtained on the Perceived Stress Scale was 17.30 (SD = 6.312). No differences were observed between men and women (t = −1.135, *p* = 0.129). A total of 58.3% of the participants indicated that they often or very often felt nervous or stressed, and 25% stated that they often or very often became angry because they felt that things were out of their control. If a score of 21 (third quartile) was considered the cut-off point, it could be said that 37.5% of the participants reported symptoms of stress, with no differences observed between men and women (χ^2^ = 2.476, *p* = 0.116).

### 3.5. Anxiety and Depressive Symptoms

The cut-off points are 4 or more for the anxiety subscale and 2 or more for the depression subscale, with higher scores indicating a more severe problem (the maximum possible being 9 in each of the subscales), and the maximum in both scales 18. The overall mean score of the scale was 7.972 (SD 4.80). On the anxiety subscale, the mean was 4.819 (SD = 3.031), and on the depression subscale the mean was 3.153 (SD = 2.504). A total of 15.3% of students obtained a score of 14 or higher on the Goldberg scale.

Considering a cut-off point on the anxiety subscale of four points or more, 54.9% of the participants are at risk of suffering from anxiety. For depression, and considering a cut-off point of two or more points, 43.4% are at risk of suffering depression. No differences were observed between men and women for suffering from anxiety (χ^2^ = 2.219, *p* = 0.136) or depression (χ^2^ = 0.152, *p* = 0.697).

### 3.6. Salivary BDNF and Cortisol Concentration

The mean BDNF level of the sample was 260.098 pg/mL (SD = 97.540) and the mean cortisol level was 0.580 pg/dL (SD = 0.281). There was no significant correlation between salivary BDNF and cortisol concentration (*p* = 0.881)

There were no differences in BDNF levels between men and women, although there were differences in cortisol levels, which were higher in women than in men (Table 2). A correlation of cortisol levels and age was observed (*p* < 0.05), as cortisol levels increase with age, but there was no relationship with BDNF levels. Differences in BDNF levels were also observed between participants who had a chronic disease or performed sports versus those who did not. Cortisol levels were also related to sport and there was a trend (*p* = 0.059) between cortisol levels and alcohol consumption, with cortisol levels higher in people who consumed alcohol (Table 2).

### 3.7. Relationships between BDNF and Cortisol Concentration in Saliva with Psychological Alterations

BDNF levels did not differ between people with normal sleep and those with poor sleep quality, but there were differences in cortisol levels and sleep quality as measured by the Pittsburg Sleep Quality Index. No relationship were observed between BDNF and cortisol levels or BDNF and stress or anxiety. There was a relationship between BDNF levels and depression, as people with higher symptoms of depression had higher BDNF levels than people without symptoms. There was also a significant correlation between cortisol levels and the Pittsburg Sleep Quality Index (r = 0.247, *p* = 0.036), as cortisol levels were higher in people with poor sleep quality (Table 3).

**Figure 1:**
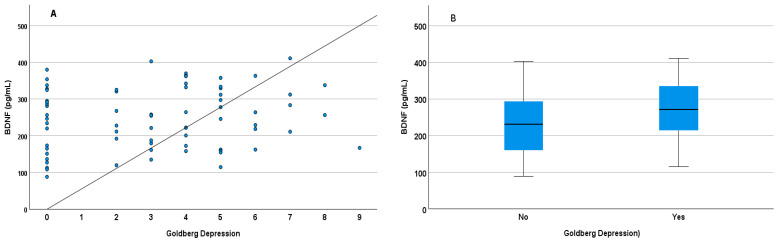
Relationship between salivary BDNF levels and Goldberg depression scale score as continuous variables (**A**) Each dot refers to the Goldberg score (depression subscale) and BDNF level as dichotomized values (**B**). All differences between groups (panel **B**) were significant (*p* < 0.05).

**Figure 2:**
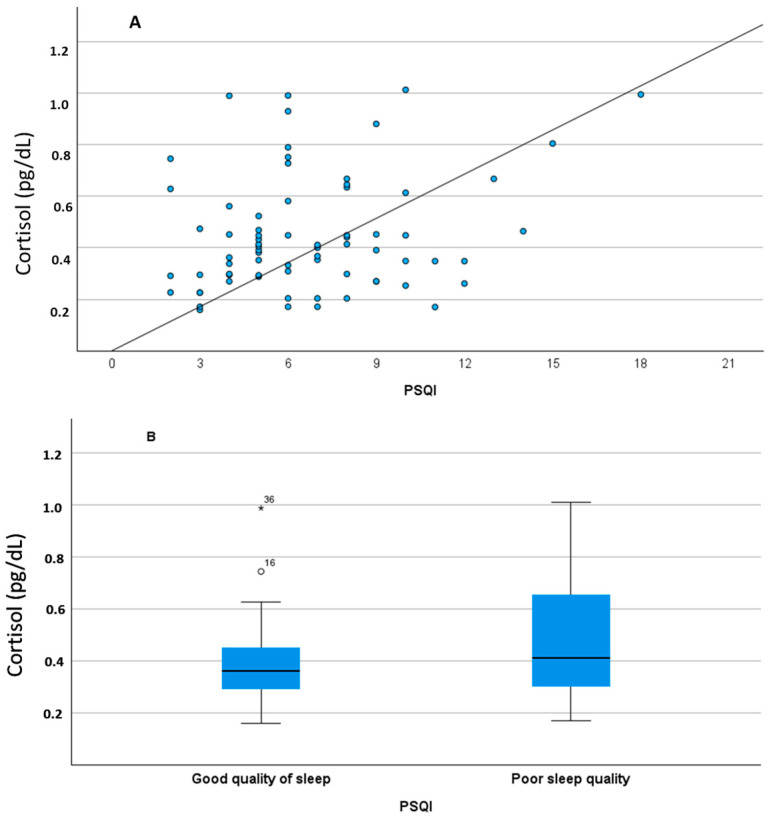
Relationship between salivary cortisol levels and Pittsburg Sleep Quality Index score as continuous variable (**A**) and as dichotomized values (**B**). All differences between groups (panel **B**) were significant (*p* < 0.05).

**Figure 3:**
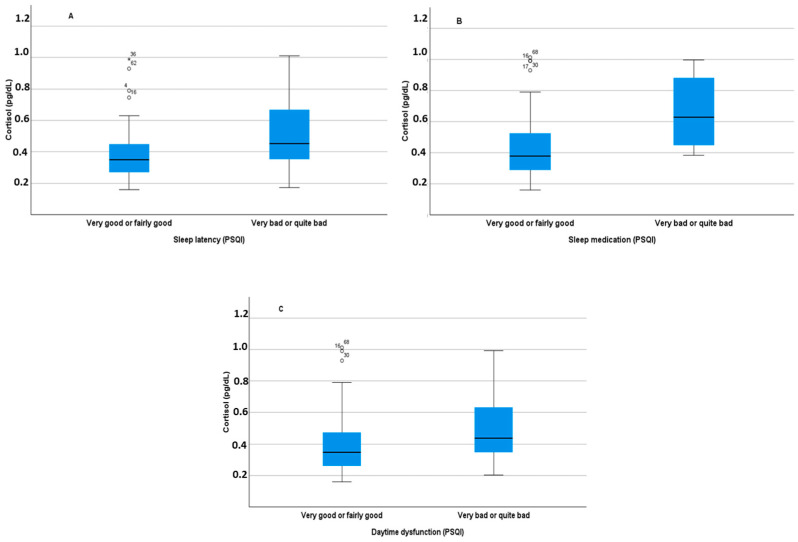
Relationship between salivary cortisol levels and Pittsburg sleep quality subdomains of sleep latency (**A**), Use of sleeping medication (**B**) and daytime dysfunction (**C**). All of the differences between groups in the three panels were significant (*p* < 0.05.).

Figure 1 shows the relationship between BDNF levels and depressive symptoms, although the correlation was not significant (Figure 1A). There was a tendency for a greater increase in BDNF levels with an increase in the Goldberg depression subscale. When the variable used was dichotomous, differences in BDNF levels were observed (Figure 1B). BDNF levels were therefore lower in people without any depressive symptoms (t = −1.978, *p* = 0.026). The relationship between cortisol levels and the Pittsburg Sleep Quality Index score is significant. Higher cortisol levels were observed in students with higher Pittsburg scores, i.e., with more sleep difficulties (r = 0.247, *p* = 0.036, Figure 2A). As seen in Figure 2B, the mean cortisol level in students manifesting good sleep quality was lower than in students indicating poor sleep quality (t = −1.857, *p* = 0.034). In addition, differences in cortisol levels were associated with certain domains of the Pittsburgh Sleep Quality Index (Figure 3A–C). In this regard, differences in cortisol levels are observed in the dimensions of sleep latency (t = −1.857, *p* = 0.034), Sleep Medications (t = −2.503, *p* = 0.007), and Daytime Dysfunction (t = −1.523, *p* = 0).

### 3.8. Multivariate Logistic Regression Models

#### 3.8.1. Explanatory Model of Depressive Symptoms

A binary logistic regression model was applied to determine the influences of the variables related to depressive symptoms. Based on the variables found to be significant in the previous analyses, sociodemographic factors (age and sex) were analyzed. Sociodemographic factors (age and sex), lifestyle (chronic illness, sport), sleep quality, cortisol, and BDNF levels were analyzed to explain and predict the depressive symptoms of the study sample. When the binary logistic regression model was performed, only the BDNF levels were included as a predictor variable (χ^2^ Hosmer-Lemeshow = 3.696 *p* = 0.883). Each unit of BDNF increased the risk of higher depressive symptoms by 1.006 (*p* < 0.046).

#### 3.8.2. Explanatory Model of Insomnia Symptoms

An explanatory model was also produced for each dimension of the Pittsburg Sleep Quality Index (PSQI) that had shown a significant relationship with cortisol levels. The variables that were significant in the bivariate analyses were included in the binary logistic regression models. These variables were sex, age, sport, alcohol consumption (AUDIT-C), Goldberg-depression score, cortisol, and BDNF levels. Alcohol consumption and BDNF levels were included. For alcohol, a trend was observed, although it was not significant (*p* = 0.059), and the BDNF level variable was included because the Pittsburg scores correlated significantly with the scores obtained on the Goldberg depression subscale (r = 0.366, *p* = 0.002). Sleep latency was related to playing a sport. People who did not exercise regularly had sleep latency that was up to six times poorer than those who did (OR = 6.001, *p* = 0.003). Furthermore, sleep latency was related to cortisol levels (OR = 15.150, *p* = 0.028). Sleep medication only appeared to be related to cortisol levels (OR = 185.142, *p* = 0.019). As with sleep latency, daytime dysfunction worsened when sports were not performed. The probability that it is bad or very bad multiplies by 2.537 when sports are not practiced (OR = 2.537, *p* = 0.08). The model fit for sleep latency was as follows: Hosmer-Lemeshow = 6.926, *p* = 0.545. The model fit for use of medication for sleeping was as follows: Hosmer-Lemeshow = 3.576, *p* = 0.893. The model fit for daytime dysfunction was as follows: Hosmer-Lemeshow = 5.465, *p* = 0.707.

## 4. Discussion

To our knowledge, this is the first study to report increased salivary BDNF concentrations in students who had more depressive symptoms according to the cut-off value on the Goldberg scale, whereas no associations were found with salivary cortisol concentrations. The high prevalence of depressive symptoms in this sample replicated recent data reported in other studies of university students in Spain [16,39,55]. Interestingly, the association was specific for depressive symptoms and not for anxiety symptoms, quality of sleep, or insomnia. As far as we know, there are no studies in the literature that have investigated the relationships between BDNF and cortisol levels in saliva with prevalent psychological alterations in students.

The results of increased BDNF contrast with previous reports that measured BDNF in serum or plasma samples, which found lower concentrations of the neurotrophic factor in the most severely depressed patients [51]. In addition, effective treatments for depression (antidepressant drugs or other non-pharmacological interventions) are able to increase BDNF levels [22,23,24,25,26,27,28,56]. In particular, several recent studies focusing on the functions of the HPA axis and brain imaging in patients suffering from burnout syndrome demonstrated that chronic work stress has a major impact in terms of HPA dysregulation, decreased BDNF, impaired neurogenesis, and atrophy of limbic structures (for a review, see Chow et al. [57]). However, no significant differences were found between BDNF and cortisol in our population. Onen Sertoz et al. [58] found no significant differences in HPA axis function when measuring cortisol in blood samples, but serum BDNF levels were significantly lower in burnout patients than in healthy controls. In contrast, a study of people with fibromyalgia found that both plasma and serum levels of BDNF were higher in patients than in healthy controls, leading to the hypothesis that BDNF is higher in patients with fibromyalgia because it participates in many compensatory modulatory mechanisms of pain [59,60]. Likewise, plasma BDNF levels were significantly higher in workers exposed to occupational stress and suffering from adjustment disorders [61], whereas no significant differences appeared in the blood cortisol levels reported in the present study. Therefore, it seems possible that the higher salivary levels of BDNF in students with more depressive symptoms were the expression of initial compensatory neuroprotective mechanisms; in fact, none of them suffered from clinically diagnosed depressive disorder, and none took antidepressant drugs. When exposure exceeds these homeostatic mechanisms in susceptible individuals, this can probably lead to serious depression, which would be associated with the depletion of neurotrophins and the deterioration of neuroplasticity until the brain atrophy that is reported in mood disorders or chronic stress occurs [21,59,62,63].

This hypothesis about the initial elevation of BDNF levels as a homeostatic mechanism is supported by some interesting preclinical evidence. For example, BDNF gene expression was reported to increase in different brain regions of rats (hippocampus, amygdala, cortex) after different types of stress (maternal separation, social defeat, acute and chronic restraint [63]), and an elevation of BDNF levels was found in rats after acute and chronic stress [64,65]. In particular, BDNF could counteract the detrimental effects of glucocorticoid excitotoxicity under stress. An interesting study by McMillan et al. [66] showed a significant increase in BDNF expression in the hippocampus of macaques in response to chronic exposure to cortisol, suggesting that this increase may protect the brain from the excitotoxic effects of high glucocorticoid levels.

Poor sleep quality is prevalent among university students in Spain and has been estimated at 49.3%, with a range that oscillates between 30.3% and 59.7% depending on the measurement methods used [55,66,67,68,69], with rates of up to 67.1% during the COVID-19 lockdown [70]. These prevalence rates among Spanish university students are consistent with those recorded in other European countries, i.e., approximately 40% [44]. Salivary cortisol was found to be elevated in students with poor sleep quality compared with those with good sleep quality, but no effects were found with self-perceived stress or anxiety and depressive symptoms. This apparently contradicts the results of previous findings, such as those by Suh [71], which showed that students who slept poorly during periods of stress had lower levels of salivary cortisol secretion in the early morning. Similar results were obtained by David et al. [72], who instructed participants to carry out the collection 30 min after waking up and encouraged them to wake up at 8 a.m. to perform the collection at 8:30 a.m. In this study, they also found that poorer sleep quality was a predictor of lower morning salivary cortisol levels 30 min after waking up. It should be considered that the circadian rhythm of cortisol secretion has a waveform pattern, with the nadir for cortisol occurring at about midnight, and its release then rises approximately 2–3 h after sleep onset and continues to rise into the early morning and early waking hours [73]. Cortisol peaks at about 9 a.m.; as the day continues, the levels decline gradually. Cortisol begins to rise rapidly upon the first-morning awakening and continues to rise for about 60 min. This phenomenon is called the awakening response. In this regard, Vargas et al. [74] reported that salivary cortisol immediately upon waking up was lower in students with poor sleep quality, but it subsequently increased to a greater extent during the cortisol awakening response. These results are similar to those obtained in the present study, in which cortisol was measured between 10 and 11 a.m. Dysfunctional HPA axis activity may play a role in some sleep disorders, but in other cases, the HPA axis dysfunction is actually the result of a sleep disorder, as seen in obstructive sleep apnea [75]. HPA axis hyperactivity can lead to fragmentation of sleep, decreased slow-wave sleep, and shortened sleep time. To complicate matters, sleep disturbances can worsen HPA axis dysfunction, thereby worsening the cycle. Both insomnia and obstructive sleep apnea are specific sleep disorders that are associated with HPA dysfunction.

This study suffers from some limitations that must be acknowledged. First, the cross-sectional design of the study and the relatively small size of the sample did not allow more subgroups of students to be created by considering whether or not they practiced sports, were overweight, or had risky alcohol consumption. All of these parameters are potentially relevant variables for understanding the relationships between BDNF and cortisol and psychological alterations. Another issue relates to the extent to which salivary BDNF levels may reflect brain BDNF concentrations. However, since BDNF can cross the blood-brain barrier [76] and changes in central and peripheral BDNF have been found to be positively correlated in rodents [77], it is likely that circulating BDNF, also measured in saliva, contributes to protecting neuronal cells and maintaining their function. Finally, other factors known to influence cortisol and BDNF levels should be taken into account, such as the presence of a circadian rhythm with significantly higher levels in the morning [34]. To minimize this bias, we chose to collect saliva samples between 10:00 and 11:00 a.m., but clearly, new studies are necessary to assess the concentrations of BDNF and cortisol in the early morning to analyze any changes in circadian rhythm that can help to understand the relationships between these two substances and the investigated psychological alterations. We should be cautious in interpreting the significance of the associations between salivary BDNF and cortisol concentrations and psychological symptoms in university students because other psychological functions, such as cognitive function, executive function, and personality variables may be involved, as demonstrated in other populations [78,79]. In the case of cortisol, certain emotion-regulation strategies, such as suppression and reappraisal, predict changes in cortisol release [80,81]. For BDNF, a connection between BDNF and executive functions and memory has been demonstrated [82,83]. Future studies should evaluate the influences of these variables on the associations found between salivary BDNF and cortisol and between depressive symptoms and sleep, respectively.

## 5. Conclusions

In conclusion, this study demonstrated higher levels of BDNF in saliva in students who had depressive symptoms, whereas this association is lacking with other common psychological symptoms in this population (anxiety, stress, and insomnia). No significant associations were found between BDNF and cortisol, and salivary cortisol was associated with poorer sleep quality and with some sleep-related subdimensions. As mentioned above, the small sample size is an important limitation of this study that affects the relevance of our findings, which should be considered as preliminary and requiring validation with larger samples. However, the results of this study suggest that BDNF could play a key role in the pathophysiology of disorders related to depressive symptoms and that an elevation of its peripheral levels could contribute to protection against the deterioration of depressive symptoms and associated disorders. The increase in cortisol suggests a need to evaluate its production rate at different times of the day in order to clarify when levels are highest in people with depressive symptoms.

## Figures and Tables

**Table 1:** Sleep quality analysis based on PSQI subdimensions.

	Good or Fairly Good	Bad or Fairly Bad
Subjective sleep quality	70.8%	29.2%
Sleep latency	58.3%	41.7%
Sleep duration	63.0%	36.1%
Habitual sleep efficiency	83.3%	16.7%
Sleep disturbances	97.2%	2.8%
Use of sleeping medication	91.7%	8.3%
Daytime dysfunction	58.3%	41.7%

**Table 2:** Salivary BDNF and cortisol concentration as functions of sociodemographic variables.

		BDNF—Mean (SD)	Cortisol—Mean (SD)
Gender	Male	230.33 (SD = 85.21)	0.31 (SD = 0.10)
	Female	254.27 (SD = 82.35)	0.48 (SD = 0.05) *
Age (correlation coefficient and *p* value)		r = 0.117 *p* = 0.326	r = 0.239 *p* < 0.05
Chronic disease	No	255.97 (SD = 83.90)	0.445 (SD = 0.23)
	Yes	208.38 (SD = 71.20) *	0.466 (SD = 0.24)
Play sport on regular basis	No	223.52 (SD = 76.87)	0.52 (SD = 0.22)
	Yes	260.62 (SD = 83.68) *	0.42 (SD = 0.22) *
Smoking habit	No	251.88 (SD = 84.13)	0.44 (SD = 0.23)
	Yes	233.24 (SD = 81.85)	0.55 (SD = 0.18)
Risky alcohol intake	No	240.50 (SD = 81.25)	0.401 (SD = 0.22)
	Yes	255.93 (SD = 84.55)	0.483 (SD = 0.22)

Significant differences are marked with an asterisk (*—*p* < 0.05).

**Table 3:** Salivary BDNF and cortisol concentration as functions of psychological alterations detected in the students.

		BDNF	Cortisol
Athens scale	Normal sleep	242.845 (SD = 82.700)	0.420 (SD = 0.218)
	Insomnia symptoms	257.334 (SD = 83.812)	0.483 (SD = 0.229)
Pittsburg Sleep Quality Index (ICSP)	Good	236.436 (SD = 80.669)	0.393 (SD = 0.176)
	Bad	257.950 (SD = 84.232)	0.485 (SD = 0.246) *
Self-perceived stress	Normal	252.009 (SD = 84.220)	0.428 (SD = 0.197)
	Symptoms of stress	245.470 (SD = 82.349)	0.475 (SD = 0.258)
Anxiety symptoms	No	240.199 (SD = 82.848)	0.408 (SD = 0.201)
	Yes	256.154 (SD = 83.339)	0.478 (SD = 0.237)
Depressive symptoms	No	232.340 (SD = 84.022)	0.435 (SD = 0.224)
	Yes	270.466 (SD = 77.679) *	0.463 (SD = 0.226)

Significant differences ared marked with an asterisk (* = *p* < 0.05).

## Data Availability

The data presented in this study are available on request for scientific purposes from the corresponding author.
